# The relationship between economic status, poverty, and health among Arabs in Israel

**DOI:** 10.1186/s13584-026-00770-3

**Published:** 2026-07-02

**Authors:** Mohammad Khatib

**Affiliations:** 1 The Galilee Society, the Arab National Society for Health Research & Services, PO Box 330, Shefa-‘Amr, 20200 Israel; 2 Zefat Academic College, Jerusalem St. 11, PO Box 160, Zefat, 13206 Israel

**Keywords:** Arabs in Israel, Health disparities, Health knowledge, Physical activity, Self-rated health, Socioeconomic status, Poverty

## Abstract

**Background:**

Arabs in Israel, comprising about 21% of the population, are recognized as an indigenous minority with full citizenship rights, yet they face persistent structural disadvantages compared to Jewish citizens. These disparities are evident across socioeconomic, educational, employment, and health domains. High poverty rates, particularly among women, children, and the older adults, are aggravated by limited governmental investment in infrastructure, education, and health services. Arab women’s labor force participation remains notably low due to systemic barriers. Extensive evidence links socioeconomic status with health outcomes and behaviors. This study examines how sociodemographic factors and health knowledge are associated with economic status, self-rated health, and health behaviors among Arabs in Israel.

**Methods:**

This cross-sectional study utilized data from the 2015–2016 Health and Environment Survey among Arabs in Israel (HESPI). Employing a three-stage stratified cluster sampling of over 2,000 households, face-to-face interviews gathered socio-demographic, economic, and health-related data. The final sample included 2,041 adults representing diverse socioeconomic and geographic groups within Arab communities.

**Results:**

Approximately half of participants lived below the poverty line (51.7%). Those with lower economic status exhibited poorer self-rated health (*p*<.001, d = 0.22), higher prevalence of chronic illness (*p*=.009, d = 0.13), and lower engagement in physical activity (*p*<.001, d = 0.29), while smoking and BMI did not significantly differ by economic status. Family size moderated the relationships between economic status and both chronic illness (*p*<.001, 95%CI = 1.33, 2.19) and SRH (*p*<.001, 95%CI = -0.33, -0.13), indicating that smaller families are less likely to experience these negative effects of low economic status. Educational attainment slightly moderated the link between economic status and physical activity (*p*=.016, η2 = 0.003). Regression analyses revealed that gender, age, education, and health knowledge were significantly associated with health outcomes and behaviors (R^2^ = 0.10 to R^2^ = 0.43), with health knowledge mediating several of these associations.

**Conclusions:**

The findings indicate that poverty is associated with poorer health outcomes and behaviors among Arabs in Israel. Smaller family size and higher education may mitigate the negative effects of poverty, and health knowledge plays a mediating role for health outcomes and behaviors. Findings suggest that socioeconomic empowerment and higher education are important in promoting health equity.

## Background

Arabs in Israel, constituting 21% of the country’s citizens, are recognized as an indigenous population with individual civil rights, yet they encounter systemic disadvantages relative to Jewish citizens [1]. These disparities are evident in multiple spheres, including socioeconomic status, employment, education, and political and social inclusion [2]. In 2021, 38.8% of Arabs in Israel lived below the poverty line, 49% among Arab children and 38.9% among Arab older adults compared to 11.9% among non-Orthodox Jews overall, 13.2% for children, and 15.3% for the older adults [3]. The persistent poverty among Arab communities is further intensified by limited government investment and underdevelopment in infrastructure, health services, and educational systems within Arab municipalities [4]. As a result, Arabs in Israel consistently report poorer health outcomes and lower self-rated health (SRH) in comparison to the Jewish majority [5].

Employment rates among Arab women in Israel are markedly lower than those of women in the general population, standing at 37% compared to 77%, respectively [4]. It is important to note that among employed Arab women, approximately 41% work part-time not by choice, but due to structural, familial, and other constraints related to the labor market [6]. These employment gaps are largely shaped by structural and systemic barriers, including residential segregation, institutional discrimination, limited access to public transportation, and insufficient childcare options. For instance, in 2020, only 8% of daycare subsidies were allocated to the Arab population, even though Arab toddlers represented 24% of all toddlers in Israel [7].

The relationship between economic status, particularly poverty, and health outcomes has been a central theme in public health research. Extensive evidence across different populations highlights that economic disparities profoundly shape health through multiple pathways, influencing both physical and mental health indicators, health behaviors, and overall well-being. Individuals and families with low income consistently face poorer health outcomes and limited access to medical services, preventive care, and health-promoting resources compared to those with higher socioeconomic status [8].

Multiple studies have documented persistent health disparities associated with economic status. A systematic review by Barakat and Konstantinidis (2023) found that individuals with high SES exhibit the best health outcomes, including better mental health and fewer cardiovascular risks, whereas low SES is associated with poorer outcomes, such as higher prevalence of mental disorders and cardiovascular mortality [8]. These disparities are reflected across multiple health indicators, including higher rates of chronic diseases, reduced life expectancy, and increased prevalence of mental health disorders. Access to healthcare is a key determinant underpinning these outcomes. Gulati and others demonstrated that disparities in healthcare access contribute to the association between low socioeconomic status and poorer health outcomes, including impacts on preventive health behaviors [9]. Individuals from low-income backgrounds often encounter multiple barriers to medical services, such as financial limitations, inadequate healthcare infrastructure in disadvantaged areas, and lack of health insurance coverage. These barriers contribute to delayed diagnoses and treatment, thereby exacerbating health conditions and diminishing overall well-being.

The link between poverty and health behaviors has also been extensively documented. Phelan, Link, and Tehranifar (2010) found that individuals from lower socioeconomic backgrounds are more likely to smoke than those with higher incomes [10]. Economic hardship and chronic stressors associated with poverty may contribute to elevated smoking rates, as smoking often functions as a maladaptive coping mechanism [11]. Economic status similarly influences physical activity. Giskes et al. (2010) reported that individuals from lower socioeconomic strata tend to have limited access to recreational facilities, safe public spaces, and affordable sports programs, resulting in lower physical activity levels and increased prevalence of obesity and related health conditions [12].

Nutritional behaviors are likewise shaped by economic constraints. Rehm et al. (2016), in their investigation of dietary intake among U.S. adults, found that individuals from low-income households often have limited access to nutritious foods due to financial barriers [13]. Consequently, they tend to consume more inexpensive, processed, and less nutritious products, which heightens their risk of obesity, diabetes, and cardiovascular disease.

This study explores whether and how sociodemographic factors influence the relationship between economic status and health outcomes, SRH, and health behaviors among Arabs in Israel. It also examines whether general or specific health knowledge mediates or moderates this relationship.

## Methods

### Study design and population

This cross-sectional study is based on data collected from the Health and Environment Survey among Arabs in Israel (HESPI), conducted by Rikaz – the Applied Social Research Center of the Galilee Society – between November 2015 and February 2016. The study population consisted of all Arabs households residing in Israel during the year 2015.

### The sample

A three-stage stratified cluster sampling design utilizing systematic random sampling was implemented. The sampling frame encompassed segregated Arab municipalities and mixed Arab–Jewish cities, and enumeration areas were classified according to the Israeli Central Bureau of Statistics (CBS) 2008 Population Census. These enumeration areas functioned as the primary sampling units (PSUs) [14].

In the first sampling stage, 75 enumeration areas were selected through systematic random sampling. In the second stage, 30 households were randomly drawn from each selected area. In the third stage, a single individual aged 18 years or older was selected from each household. Inclusion criteria were family members aged 18 years and older, both males and females, residing permanently in the same household. Family members who reside permanently outside the household were excluded. From this age-ordered family list, participants were selected using Kish selection Tables [15, 16], to participate in in-depth interviews.

The sample size was determined to provide sufficient statistical power for comparing participants with and without one or more chronic conditions. Based on an earlier reported prevalence of 14.5% for the presence of one or more chronic conditions, the sample size was estimated at 2,250 households [17].

### Data collection

The research instrument was an anonymous questionnaire, and systematic sampling was used to recruit households within each enumeration area until 30 households were reached. A household member aged 18 years or older was selected for interview. If the selected individual was unavailable, interviewers made one or two additional visits to complete the questionnaire. Quality control procedures included review of completed questionnaires, field reports, and interviewer comments by the field coordinator.

Of 2,246 sampled households, 2,018 participated in the study (response rate: 89.8%). Data was collected about total of 9,063 individuals (all families members), and 2,018 selected adults aged 18 or older (971 men and 1,047 women) completing in-depth follow-up interviews. Prior to conducting the interviews and completing the questionnaire, participants were asked to sign an informed consent form after being presented with the details of the study, including the assurance of questionnaire anonymity.

### The questionnaire

A socio-demographic questionnaire was developed ad hoc for the present study. It included variables such as gender, age, educational attainment, geographical district, and household income. Income data was used to determine whether the participant classified as below or above the national poverty line^1^. The participant was considered below the poverty line if his\her total net monthly income was less than 2,526 NIS, according to the official poverty threshold for 2015 [18].

A health-related questionnaire was also constructed ad hoc and included items assessing selected health behaviors (e.g., cigarette and waterpipe (Nargila) smoking, physical activity), self-assessed health knowledge, presence of chronic diseases, as well as anthropometric measures (weight and height). In addition, participants were asked to provide a Self-Rated Health (SRH) score [19]. 

### Participants

The study included 2,041 Arab adults in Israel, comprising 990 men (48.5%) and 1,051 women (51.5%). Participants ranged in age from 18 to 94 years (M = 41.0 years, SD = 16.10). The majority were Muslim (81.7%), resided in the northern district (72%), and lived in Arab municipalities (87%). locality size distribution was as follows: large localities (53%), medium-sized localities (27%), and small localities (20%). Most participants were married (77%), with an average household size of 4.0 people (SD = 2.06). Educational attainment varied: less than secondary education (38%), secondary education (26%), high school matriculation certificate (23%), and academic education (12%). Detailed demographic characteristics are presented in Table 1.

Participants living below the poverty line differed significantly from those above the poverty line across multiple demographic characteristics. Those below the poverty line were older, had larger households, and a higher proportion were widowed. They also had lower educational attainment and lower employment rates. Additionally, a higher proportion of participants below the poverty line were Muslim and resided in southern Israel compared to those above the poverty line.

**Table 1:** Demographic characteristics of the participants* (*N* = 2041)

Characteristic	Total sample	Below poverty line (*n* = 908)	Above poverty line (*n* = 847)	Difference
Age (years) M (SD) (range: 18–94)	40.90 (16.10)	41.64 (16.70)	38.59 (14.22)	**t(1738.88) = 4.12**,*p*<.001
Family size M (SD) (range: 1–14)	4.32 (2.06)	4.68 (2.23)	4.22 (1.76)	**t(1706.66) = 4.74**, *p*<.001
Gender n (%)				
Female	1051 (51.5)	477 (52.5)	416 (49.1)	Z = 1.43 *p* = .152
Male	990 (48.5)	431 (47.5)	431 (50.9)	
Age categories n (%)				
18–24	314 (15.4)	137 (15.1)	144 (17.0)	**χ** ^**2**^ **(4) = 26.45** *p* < .001
25–34	527 (25.8)	208 (22.9)	252 (29.8)	
35–44	480 (23.5)	245 (27.0)	181 (21.4)	
45–59	415 (20.4)	176 (19.4)	185 (21.8)	
60+	305 (14.9)	142 (15.6)	85 (10.0)	
Marital status n (%)				
Single	303 (15.0)	142 (15.7)	133 (15.9)	**χ** ^**2**^ **(3) = 37.80** *p* < .001
Married, engaged	1552 (76.7)	665 (73.7)	679 (81.0)	
Separated, divorced	40 (1.9)	23 (2.5)	8 (1.0)	
Widowed	129 (6.4)	72 (8.0)	18 (2.1)	
Education level n (%)				
Less than secondary	776 (38.4)	405 (45.3)	234 (27.7)	**χ** ^**2**^ **(3) = 146.93** *p* < .001
Secondary certification	521 (25.8)	261 (29.2)	191 (22.6)	
High school, matriculation certificate	472 (23.4)	182 (20.4)	233 (27.6)	
Academic	250 (12.4)	46 (5.1)	187 (22.1)	
In labor force n (%)	1066 (52.9)	418 (46.6)	555 (66.3)	**Z = 8.26** *p* < .001
Employed from labor force n (%) (from *n* = 1066)	975 (91.5)	359 (85.9)	541 (97.5)	**Z = 6.80** *p* < .001
Total employment n (%) (from *n* = 2041)	975 (48.4)	359 (40.0)	541 (64.6)	**Z = 10.25** *p* < .001
Religion n (%)				
Muslim	1663 (81.7)	792 (87.4)	624 (73.9)	**χ** ^**2**^ **(2) = 55.62** *p* < .001
Druze	241 (11.8)	82 (9.1)	132 (15.6)	
Christian	131 (6.5)	32 (3.5)	88 (10.4)	
District in Israel n (%)				
North	1477 (72.4)	634 (68.7)	664 (78.4)	**χ** ^**2**^ **(2) = 27.15** *p* < .001
Center	290 (14.2)	118 (13.0)	98 (11.6)	
South	274 (13.4)	166 (18.3)	85 (10.0)	
Type of residence n (%)				
Arab	1778 (87.1)	798 (87.9)	734 (86.7)	Z = 0.77 *p* = .441
Mixed Jewish-Arab	263 (12.9)	110 (12.1)	113 (13.3)	
Size of residence n (%)				
Large, over 15,000	1086 (53.2)	472 (52.0)	447 (52.8)	χ^2^(2) = 5.61 *p* = .061
Medium, 5000 to 15,000	544 (26.7)	222 (24.4)	236 (27.9)	
Small, less than 5000	411 (20.1)	214 (23.6)	164 9.4)	

Bold values: *p* < .001

*Percentages were calculated excluding missing data

### Variables and measures

*Health Status Indicators*:Body Mass Index (BMI) was calculated from self-reported height and weight. Chronic illness was measured as a dichotomous variable (yes/no). Medication use was assessed as a categorical variable with three levels: daily, occasionally, and none.

*Health Perception:* Overall health perception was assessed using two items measured on a 5-point scale: (1) SRH (1 = very bad to 5 = very good) and (2) satisfaction with health status (1 = very low to 5 = very high). The items were strongly correlated (r = .85, p < .001), and mean scores were calculated to create a composite measure [20].

*Health Behaviors:* Physical activity was measured as a dichotomous variable (yes/no). Smoking status was assessed using dichotomous variables for cigarette smoking and/or nargileh use (yes/no).

*Health Knowledge:* General health knowledge: Eight items were measured on a 5-point scale (α = .95). Mean scores were calculated, with higher scores indicating greater health knowledge. Perceived nutrition knowledge was assessed by the question "do you have information about healthy nutrition?" and using dichotomous variable (yes/no).

*Economic Status:* Dichotomous variable indicating individual income below or above the 2015 Israeli poverty line threshold.

Sociodemographic variables included gender, age, household size, marital status, educational level, employment status, religion, geographic district, type of locality (Arab municipality/mixed Arab-Jewish city), and locality size.

### Data analysis

Data was analyzed with SPSS software ver. 28. First, 286 participants did not provide information about their economic status. An examination of their health perception, health behaviors, and knowledge, revealed that they had a lower health related status than those who provided information about their economic status. A higher percentage of them reported a chronic illness (*p* < .001), and their general perception of health was lower (*p* < .001). Further, a lower percent of them reported having knowledge about nutrition (*p* < .001).

Descriptive statistics were used to characterize the demographic profile of participants and the study’s dependent variables. Group comparisons (below/above the poverty line) were calculated using t-tests, chi-square tests, and Z-ratios for the significance of differences between two independent proportions. Next, the moderating role of demographic variables in the relationships between economic status and the study’s dependent variables was examined using (a) multiple linear regressions with interactions for continuous dependent variables, (b) multiple logistic regressions with interactions for dichotomous dependent variables and continuous demographic variables, and (c) analyses of variance for dichotomous dependent variables and categorical demographic variables. Significant interactions were interpreted using simple slopes and estimated marginal means. The study model was first examined using a series of multiple linear and logistic regressions for SRH and related behaviors, including demographic variables, economic status, and health-related knowledge. Mediation was examined using a series of PROCESS models (Hayes, 2022), specifically Model 4, with 5,000 bootstrap samples and 95% confidence intervals [21]. The significance level was set at *p* < .01 due to sample size.

## Results

### Differences by economic status

Approximately half of the participants were classified as living below the poverty line (n = of 1755; 51.7%), while the remainder were above the poverty line (*n* = 847; 48.3%). Table 2 shows the distribution of study variables for the total sample and stratified by economic status. The mean BMI for the sample was 26.5, with no significant differences observed between economic groups. Approximately 40% of participants had a BMI in the normal range, another 40% were overweight, and around 17% were classified as obese.

A higher proportion of participants below the poverty line reported having a chronic illness compared to those above the poverty line (34% vs. 28%, respectively). Most of the participants in both groups adhered to daily medication for their chronic condition. SRH was lower among participants below the poverty line (M = 3.78) compared to those above the poverty line (M = 4.01). Similarly, a lower percentage of participants below the poverty line engaged in physical activity (20% vs. 33%). No significant group differences were detected for smoking status. Both general health knowledge (M = 2.65 vs. M = 2.87) and perceived nutrition knowledge (49% vs. 64%) were lower among participants living below the poverty line.

**Table 2:** Health perception, related behavior, and knowledge, for the whole sample (N = 2041), and by economic status (N = 1755)

		Total sample	Below poverty line(n = 908)	Above poverty line(n = 847)	Difference
**Chronic illness, overweight:**					
BMI	M (SD), range	26.55 (4.57)(16-63)	26.64 (4.68)	26.40 (4.54)	t(1687) = 1.05 p = .294
BMI categories, n (%)	Underweight(<18)	17 (0.9)	8 (0.9)	8 (1.0)	χ^2^(4)=1.91 p = .752
	Normal(18-24.9)	758 (38.5)	330 (37.8)	326 (40.0)	
	Overweight(25-29.9)	851 (43.2)	386 (44.2)	340 (41.7)	
	Obese(30-34.9)	258 (13.1)	109 (12.5)	110 (13.5)	
	Morbid obesity (35+)	84 (4.3)	40 (4.6)	32 (3.9)	
Chronic illness	Yes, n (%)	668 (32.7)	309 (34.0)	239 (28.2)	**Z** = 2.63 *p* = .009
Daily medication for chronic illness, n (%)(of n=485)	Yes	442 (91.1)	217 (91.9)	140 (88.1)	Z = 1.29 p = .197
	Irregularly, no	43 (8.9)	19 (8.1)	19 (11.9)	
**Health perception: **					
Total SRH, n (%)	M (SD), range	3.85 (1.06)(1-5)	3.78 (1.09)	4.01 (0.98)	**t(1750.60) = -4.69 ** p < .001
**Health related behavior:**					
Physical activity	Yes, n (%)	524 (25.8)	183 (20.3)	278 (32.9)	**Z = 5.98** p < .001
Smoking (cigarettes or Nargila)	Yes, n (%)	672 (33.3)	294 (32.7)	303 (36.1)	Z = 1.48 p = .139
**Health related knowledge:**					
General health knowledge	M (SD), range	2.74 (0.99)(1-5)	2.65 (0.95)	2.87 (1.01)	**t(1709.53) = -4.73** p < .001
Perceived nutrition knowledge	Yes, n (%)	1106 (54.5)	442 (49.1)	536 (63.6)	**Z = 6.11** p < .001

Bold values: *p* < .001

### Differences by economic status and the demographic variables

Exploratory attempts were calculated to assess whether the relationships between economic status and health were moderated by the demographic variables. Two significant results were found: for family size and level of education.

Family size was found to moderate the relationship between economic status and having a chronic illness (B = 0.54, SE = 0.13, *p*<.001, OR = 1.71, 95%CI = 1.33, 2.19). Interpretation of the significant interaction with simple slopes has revealed that for smaller families, being an individual above the poverty line was related with lower odds for having a chronic illness (effect = -0.82, t = -5.57, *p*<.001), while the relationship was not significant for larger families (effect = 0.25, t = 1.36, *p*=.175).

Further, family size was found to moderate the relationship between individual economic status and perception of health (B = -0.23, SE = 0.05, β = − 0.13, *p*<.001, 95%CI = -0.33, -0.13). Interpretation of the significant interaction with simple slopes has revealed that for smaller families, being above the poverty line was related with better SRH (effect = 0.51, t = 7.47, *p*<.001), while the relationship was not significant for larger families (effect = 0.05, t = 0.70, *p*=.487).

Level of education was found to moderate the relationship between economic status and physical activity (F(1, 1727) = 5.86, *p*=.016, η^2^ = 0.003). Interpretation of the significant interaction with estimated marginal means has revealed that for more educated participants (with a full high school education or a college degree), being above the poverty line was related with higher odds for doing physical activity (F(1, 1727) = 17.55, *p*<.001, η^2^ = 0.010), while the relationship was not significant for less educated participants (with less than a full high school education) (F(1, 1727) = 2.35, *p*=.125, η^2^ = 0.001).

That is, smaller families were less likely to experience these negative effects of low economic status regarding chronic illness and the perception of health. Level of education slightly moderated the link between economic status and physical activity.

### The study model

Multiple linear and logistic regression analyses were performed to examine associations SRH and related behaviors. Independent variables included economic status and health-related knowledge, while gender (coded as 1 = male, 0 = female), age, family size, and educational attainment (coded as 1 = completion of full high school education or a college degree, 0 = less than full high school education) were included as covariates. General health knowledge was hypothesized to be associated with all outcome variables; perceived nutrition knowledge was hypothesized to be associated with BMI and chronic illness and was entered accordingly into the relevant regression models. Due to insufficient variance, daily medication adherence for chronic illness was excluded from the analyses.

Results in Table 3 show that all five models are significant. About 10% of the variance in BMI were explained by the study variables, such that for females, younger participants, and with greater health related knowledge, BMI was lower. BMI was unrelated with perceived nutrition knowledge. About 43% of the variance in the likelihood of chronic illness were explained by the study variables, such that the odds for a chronic illness were higher for older participants, living in a smaller family, and for participants with less than a full high school education. Health related knowledge and perceived nutrition knowledge were unrelated with the odds for a chronic illness. About 33% of the variance in health perception were explained by the study variables, such that for younger participants, participants with a full high school education or a college degree, and with greater health related knowledge, SRH was better. Further, about 17% of the variance in the likelihood of physical activity were explained by the study variables, such that the odds for physical activity were higher for males, younger participants, participants with a full high school education or a college degree, participants whose economic status was above the poverty line, and with greater health related knowledge. Finally, about 37% of the variance in the likelihood of smoking were explained by the study variables, such that the odds for smoking were higher for males, slightly higher for younger participants, and with lower health related knowledge. It should be noted that the high odds for smoking, regarding gender, reflect the fact that 57% of the men were smoking, compared with 11% of the women.

**Table 3:** Multiple linear and logistic regression models for SRH and related behavior, with economic status and health related knowledge (*N* = 1755)

	BMI β (*p*)	Chronic illness OR (*p*) (95%CI)	SRH β (*p*)	Physical activity OR (*p*) (95%CI)	Smoking OR (*p*) (95%CI)
Gender	**0.07 (0.003)**	0.92 (0.545) (0.72, 1.19)	0.04 (0.070)	**1.67 (< 0.001)** **(1.32**,** 2.11)**	**13.39 (< 0.001)** **(10.25**,** 17.50)**
Age	**0.22 (< 0.001)**	**1.09 (< 0.001)** **(1.08**,** 1.11)**	**− 0.48 (< 0.001)**	**0.97 (< 0.001)** **(0.96**,** 0.98)**	**0.99 (0.008)** **(0.98**,** 0.997)**
Family size	− 0.06 (0.024)	**0.91 (0.006)** **(0.85**,** 0.97)**	0.04 (0.073)	1.03 (0.337) (0.97, 1.10)	1.04 (0.275) (0.97, 1.10)
Education level	− 0.04 (0.096)	**0.66 (0.006)** **(0.49**,** 0.89)**	**0.09 (< 0.001)**	**1.96 (< 0.001)** **(1.53**,** 2.52)**	0.85 (0.257) (0.65, 1.12)
Poverty line	0.01 (0.679)	0.96 (0.759) (0.74, 1.25)	0.03 (0.109)	**1.54 (< 0.001)** **(1.21**,** 1.97)**	1.25 (0.077) (0.98, 1.61)
General health knowledge	**− 0.13 (< 0.001)**	0.89 (0.101) (0.77, 1.02)	**0.15 (< 0.001)**	**1.38 (< 0.001)** **(1.22**,** 1.56)**	**0.63 (< 0.001)** **(0.55**,** 0.72)**
Perceived nutrition knowledge	0.01 (0.829)	0.98 (0.914) (0.75, 1.30)	--	--	--
	Adj.R^2^ = 0.10	R^2^ = 0.43	Adj.R^2^ = 0.33	R^2^ = 0.17	R^2^ = 0.37
	F(7, 1642) = 26.37 *p*<.001	χ^2^(7) = 621.51 *p*<.001	F(6, 1718) = 144.94 *p*<.001	χ^2^(6) = 217.72 *p*<.001	χ^2^(6) = 536.20 *p*<.001

For logistic regressions- Nagelkerke’s R^2^

Bold values: *p* < .001

Bold vakues: P value<0.01

In light of these results (Table 3), and the results shown in Table 2, mediation was likely for BMI, total SRH, physical activity, and smoking - with general health knowledge as the mediator. It was examined with the Process procedure, model no.4, for continuous and dichotomous outcomes, controlling for gender, age, family size, and level of education. All four mediation models were found significant, with the indirect effects shown in Table 4.

**Table 4:** The indirect mediation effects for BMI, SRH, physical activity, and smoking, with general health knowledge (*N* = 1755)

	Indirect effect	SE	95%CI
BMI	-0.07	0.03	-0.15, -0.01
SRH	0.02	0.01	0.01, 0.04
Physical activity	0.04	0.02	0.01, 0.08
Smoking	-0.06	0.02	-0.11, -0.02

Bold values: *p* < .001

**Fig. 1:**
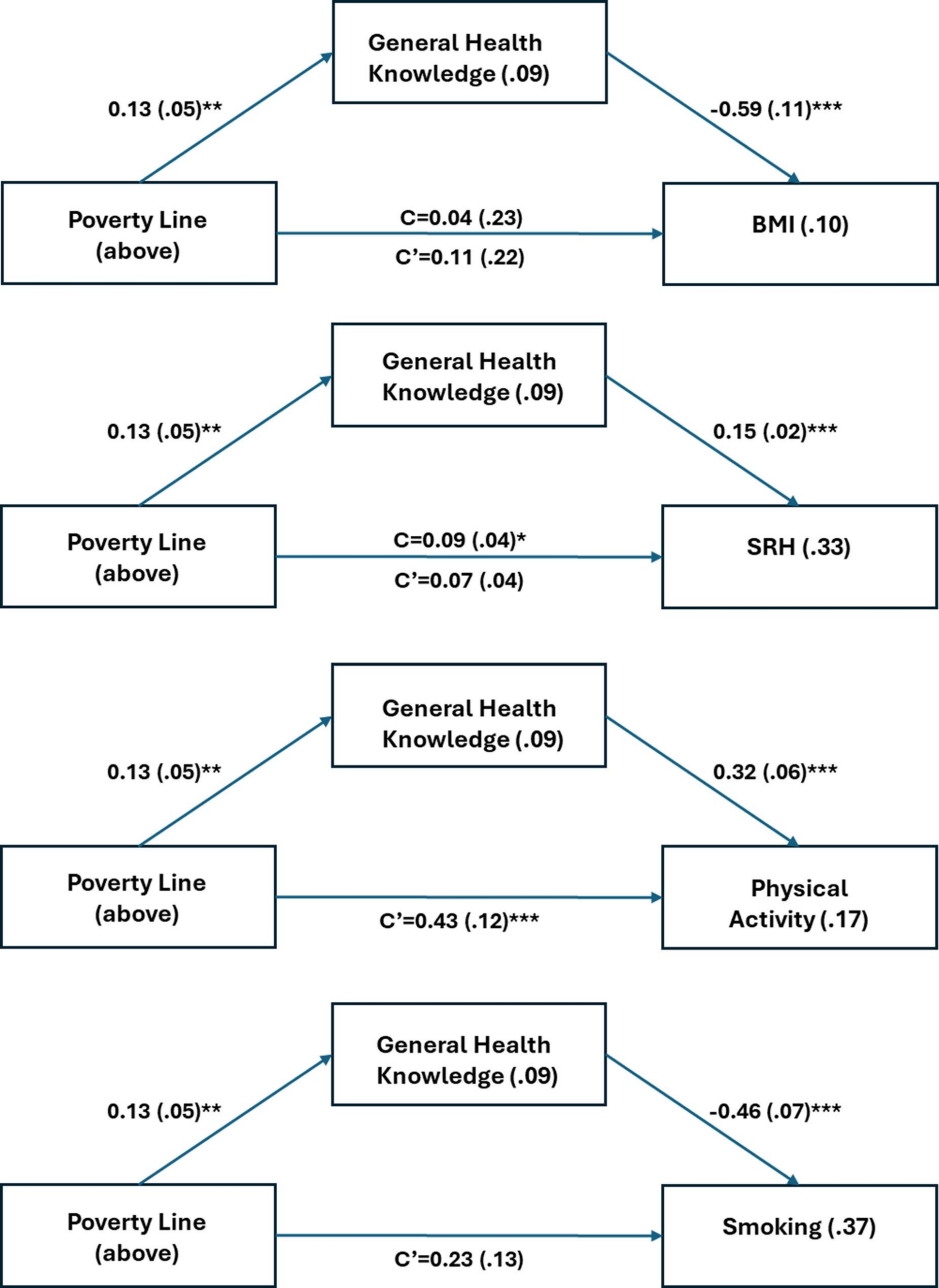
The mediating role of general health knowledge in the relationship between economic status and BMI, SRH, physical activity, and smoking. Values on arrows: B(SE), values within rectangles: R2 / Negelkerke’s R2, C = total effect for continuous outcomes, C’ = direct effect. *p<.05, **p<.01, ***p<.001

Figure 1 presents the mediated relationships. As may be observed, being above the poverty line was related to higher health related knowledge, which in turn was related to lower BMI, with a better SRH, with higher odds for physical activity, and with lower odds for smoking.

## Discussion

This study examined the relationship between economic status, health status, and health behaviors among the Arab population in Israel, with a particular focus on mediating and moderating mechanisms. The findings reveal a complex picture of health inequities shaped by multiple sociodemographic factors. The fact that more than half of the participants live below the poverty line reflects the harsh socioeconomic reality of the Arab population in Israel, as documented in official state reports by national institutions.

The findings indicate several associations between the individual economic status and key health indicators. The higher prevalence of chronic conditions and lower SRH among participants living below the poverty line is consistent with the international literature on the social determinants of health. Numerous studies have shown that poverty constitutes a major risk factor for chronic morbidity through multiple mechanisms, including exposure to chronic stress, neglect of health needs, limited access to high-quality health services, and risk behaviors [22–24].

In the context of minority groups, the literature points to heightened severity of health inequities resulting from the accumulation of multiple structural barriers. Studies of ethnic minorities in the United States, the United Kingdom, and Australia report similar patterns, in which low socioeconomic status is combined with linguistic, cultural, and access barriers, generating a substantial burden of health distress [25]. Among the Arab population in Israel, this situation is further exacerbated by geographic concentration in the periphery, the low socioeconomic ranking of most Arab localities, and cultural–linguistic barriers within the health system [26–28]. Previous studies on the health of the Arab population in Israel have documented substantial gaps in key health indicators compared to the Jewish population, including shorter life expectancy, higher infant mortality, and increased prevalence of chronic diseases [29, 30]. The present findings may contribute to this understanding by pointing to possible mechanisms through which economic status is associated with health in this population.

Contrary to expectations and to some of the existing literature, no significant differences in body mass index (BMI) were found between participants living above and below the poverty line. This result deviates from the common pattern observed in many high‑income countries, where poverty is typically associated with obesity, particularly among women and children. Several possible explanations may account for this finding. First, it may reflect poverty and nutrition paradox characteristic of societies undergoing nutritional transition, in which inexpensive, energy‑dense foods are widely available even to low‑income groups, resulting in high obesity rates across socioeconomic strata. Second, the official definition of the poverty line may not adequately capture the economic reality of Arab society, in which families classified as above the poverty line may still experience substantial economic hardship relative to the cost of living. Third, cultural factors and social norms regarding diet and body weight may outweigh the direct impact of economic status. Finally, currently, BMI is no longer considered a comprehensive measure of health status on its own, and the use of additional indicators might have changed the result.

At the same time, the findings indicate a rather high prevalence of overweight and obesity in the sample as a whole (about 60%). This pattern is alarming in view of the well-known links between excess body weight and chronic diseases such as diabetes, hypertension, and cardiovascular disease. Poor nutritional status was found to be associated with low health knowledge irrespective of economic status, suggesting the importance of health education as a potential strategy to improve this situation.

The finding that only one fifth of participants living below the poverty line engaged in physical activity, compared with one third of those above the poverty line, provides evidence of a gap in one of the preventive health behaviors. Physical activity is widely recognized as a protective factor against a range of chronic diseases and as a contributor to both physical and mental health. The observed gap is consistent with international findings indicating that populations experiencing economic hardship are less likely to engage in structured physical activity [31]. This disparity may be explained by multiple barriers, including lack of leisure time due to long working hours or multiple roles in large families, limited availability of appropriate facilities and public spaces in many Arab localities, the financial costs associated with gym memberships or equipment, and cultural barriers, particularly for women. Previous studies on the Arab population in Israel have shown that social norms and restrictions on women’s free movement in public spaces constitute a barrier to physical activity, especially in more conservative communities [32, 33].

One of the noteworthy findings of this study is the absence of significant differences in smoking prevalence between groups defined by economic status, a pattern that diverges from that observed in many high‑income countries, where smoking is more prevalent among low socioeconomic groups. This result may reflect the normalization of smoking across economic strata in Arab society, especially among men, a phenomenon documented in previous research. The particularly high smoking prevalence among Arab men in Israel has been repeatedly reported and is recognized as a key risk factor for cardiovascular disease and cancer [34]. The fact that smoking was associated with low health knowledge rather than with economic status suggests that it may represent a culturally embedded behavior that may not be directly associated with material resources. This finding supports the need for culturally tailored smoking‑cessation interventions that focus on changing social norms and increasing awareness of health risks.

The mediation analyses revealed small effects. Still, they showed that health knowledge may act as a mediator in the associations between economic status and nutritional status, SRH, physical activity, and smoking. This means that some portion of the association between poverty and health is explained by differences in health knowledge. That is, individuals experiencing economic hardships may have lower levels of health knowledge, which in turn is negatively associated with their health status and health behaviors. This finding is consistent with theoretical models of health literacy, which emphasize the importance of the ability to obtain, process, and understand basic health information for making informed health decisions [35, 36]. Populations in economic distress tend to have lower health literacy due in part to lower educational attainment, limited access to high‑quality information sources, and language and cultural barriers [37]. In the Israeli context, these barriers may be exacerbated by the relative scarcity of health‑education materials in Arabic, the limited number of Arabic‑speaking health professionals, and digital gaps that constrain access to online information [27, 29]. The practical implication of this finding is that improving health knowledge may serve as an intervention strategy for reducing health disparities. Culturally adapted health education programs delivered through community institutions such as clinics, schools, and religious and cultural centers may contribute to improving health behaviors and health status even among populations facing economic hardships.

At the same time, the findings point to the role of two demographic factors: family size and educational attainment. The study shows that the association between economic status, chronic morbidity, and health perception is significant among small families and not among large families. This finding may point to a complex role of family structure. In small families, living above the poverty line may serve as a health advantage, perhaps due to more favorable distribution of economic resources among fewer family members.

The association between economic status and physical activity was observed only among participants with a completed secondary education or an academic degree and had a very small effect. It may suggest that higher education may serve as a cultural resource that enables the translation of economic advantage into health‑promoting behavior. Higher education often provides knowledge about the importance of physical activity as well as organizational skills, the ability to access information and resources, and an orientation toward health and quality of life. This result is consistent with international evidence showing that education is a powerful determinant of health behaviors, sometimes even stronger than income [8, 24]. In the Israeli context, educational levels in the Arab population have increased substantially in recent decades, yet large gaps remain compared to the Jewish population, particularly in rural areas and among women in more conservative communities [26].

Taken together, the findings of this study call for careful attention to the structural and systemic barriers faced by the Arab population in accessing quality health services. Although Israel is characterized by universal health insurance under the National Health Insurance Law, substantial gaps exist in the realization of formal entitlements. First, co‑payments for medications, tests, and treatments constitute a major financial barrier for low-income families. For a family with several members suffering from chronic conditions, monthly co‑payments amounting to significant sums may be unaffordable, leading to treatment avoidance or non‑adherence to prescribed medications [38].

Second, supplemental health insurance, which covers services and tests beyond the basic national basket, is less prevalent among the Arab population due to its cost. Documented gaps in the uptake of supplemental insurance between the Arab and Jewish populations constitute an important source of inequality in access to advanced treatments, diagnostic technologies, and complementary health services [39]. Third, the physical availability of health services is limited in many Arab localities, particularly in peripheral regions. Shortages of specialty clinics, diagnostic centers, and mental‑health services within these localities necessitate travel to urban centers, imposing financial and time costs that many low‑income families cannot afford. In addition, the limited number of Arabic‑speaking professionals across health disciplines and the scarcity of culturally adapted informational materials impede effective communication and understanding of medical instructions.

Fourth, preventive and health‑promotion services, such as healthy‑nutrition workshops, smoking‑cessation programs, and community based physical activity initiatives are often less available or less culturally tailored in Arab communities. The finding of lower health knowledge and lower perceived nutritional knowledge among participants living below the poverty line points to the need for investment in community-based health education programs.

### Study limitations

This study was conducted about a decade ago and thus does not reflect recent changes in Arab society, such as declining fertility and family size, and partial improvements in poverty and education, which could weaken their mediating effects on health. Furthermore, rising chronic morbidity and population aging may exacerbate this relationship; therefore, updated studies are needed to assess the current validity of the association between socioeconomic status and health. Additionally, the Arab society in Israel encompasses substantial heterogeneity across its major subgroups—Muslims, Christians, Druze, and Bedouins—which likely influences the observed relationship between socioeconomic status and health outcomes found in this study.

This variability suggests that the aggregated analysis may mask subgroup-specific patterns, underscoring the need for future studies to disaggregate these groups for a more nuanced understanding of the underlying mechanisms.

The cross‑sectional design does not allow for causal inferences, and bidirectional or reverse relationships may exist; for example, poor health may lead to economic distress through reduced work capacity and increased treatment costs.

Reliance on self‑reported measures (such as chronic disease and SRH) may introduce recall bias or social desirability bias, although such indicators are widely accepted and validated in health research. Further, our use of questionnaires that have no full psychometric validation may introduce unknown measurement error. Hence, further validation studies are warranted.

The study did not include detailed information on specific types of chronic diseases, which could have provided deeper insights into the associations under investigation. Additional variables that may contribute to explaining the observed variance were not measured, such as social support, stress levels, and access to specific services.

One limitation of this study is the reliance on BMI as the sole measure of nutritional status, which overlooks critical aspects of body composition such as fat distribution, muscle mass, and individual variations related to age, sex, and ethnicity. This approach may lead to misclassification of participants’ nutritional health, particularly for athletes or older adults who exhibit normal BMI despite elevated body fat or sarcopenia. Future research should incorporate specific chronic conditions based on documented medical diagnoses rather than participants’ self-reports. Additionally, such studies should include further variables that may contribute to the associations examined here, such as social support, mental health, and the availability and accessibility of specific healthcare services. In addition, future research should incorporate complementary metrics like waist circumference or bioelectrical impedance analysis for a more comprehensive assessment.

### Conclusion and recommendations

This study provides comprehensive empirical evidence for the complex relationship between economic status, health status, and health behaviors in the Arab population in Israel. The findings indicate substantial health inequities shaped by multiple mechanisms, including health knowledge, family size, and educational attainment. Identifying health knowledge as a mediating factor suggests the potential of educational interventions to reduce health gaps.

Considering the findings, several practical recommendations emerge. First, culturally adapted health education programs should be developed and implemented through community institutions such as schools, cultural centers, and community‑based health services. Second, the availability of health services in Arab localities should be expanded, and the number of Arabic‑speaking professionals in various health disciplines should be increased. Third, the financial burden of co‑payments should be reduced, and access to supplemental health insurance should be improved. Fourth, health‑promoting infrastructures and environments should be developed in Arab communities, including culturally appropriate facilities for physical activity, particularly for women, walking paths, green spaces, and parks. Fifth, culturally tailored smoking‑cessation interventions should be designed and implemented. Sixth, policies aimed at reducing socioeconomic ranking gaps between Arab and Jewish localities and at improving physical and social infrastructures should be advanced. Future research should examine the effectiveness of existing interventions, deepen understanding of the cultural and structural determinants of health behaviors, and assess the extent to which improving health knowledge and health literacy through targeted interventions leads to sustained improvements in health indicators and health behaviors over time.

## Data Availability

The datasets used and/or analyzed during the current study are available from the corresponding author on reasonable request.

## Abbreviations

SRH: Self-rated health - SRH
HESPI: Health and environment survey of Palestinian citizens of Israel
CBS: Central bureau of statistics
PSU: Preliminary sampling units
BMI: Body mass index

## Footnotes

1 The poverty line in Israel in 2015 was 3,158 New Israeli Shekels, while the poverty line per standard person was 2,526 NIS (half of the median income), since according to the equivalence scale, one person is counted as 1.25 standard persons.
